# Artificial intelligence in bioanalytics: implications for research and education

**DOI:** 10.1007/s00216-026-06806-6

**Published:** 2026-09-09

**Authors:** Karsten Rebner, Jörg Mittelstät, Ralf Kemkemer, Günther Proll

**Affiliations:** School of Life Sciences, Reutlingen University, Alteburgstrasse 150, 72762 Reutlingen, Germany

**Keywords:** Artificial intelligence, Education, Didactic strategies, Grey-box modelling, Cell viability monitoring

## Abstract

Artificial intelligence (AI) is increasingly shaping bioanalytical research by supporting the analysis of complex, high-dimensional and multimodal datasets. Although AI-based methods often achieve remarkable predictive performance, their scientific relevance ultimately depends on the extent to which model outputs can be linked to biologically, chemically, and physically meaningful processes. This article discusses the implications of AI-supported bioanalytics for both research and higher education, with particular emphasis on model transparency, interpretability, and analytical validity. Black-box, grey-box, and white-box models are considered as distinct yet complementary approaches to knowledge generation. In this context, grey-box and hybrid modelling strategies are identified as especially suitable for bioanalytical applications because they integrate data-driven learning with established domain knowledge. The discussion is illustrated by a case study on UV/Vis spectroscopic monitoring of mammalian cell viability, demonstrating how physics-informed AI, experimental design, spectral analysis, and rigorous model validation can contribute not only to reliable predictions but also to a more mechanistic interpretation of bioanalytical data. From an educational perspective, the integration of AI extends beyond the acquisition of technical skills and requires curricula that foster critical reasoning, uncertainty assessment, and the reflective evaluation of model results. Responsible use of AI in bioanalytics therefore requires its treatment as a tool that complements scientific judgement rather than replacing analytical expertise.

**Graphical abstract:**

**Figure:**
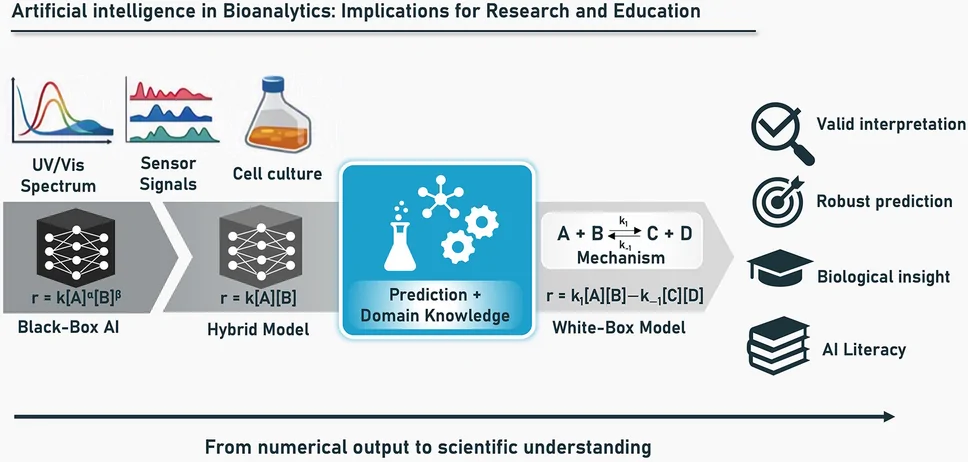

## Introduction

Bioanalytics is increasingly shifting from hypothesis-driven, low-throughput experiments to large-scale data collection, integration, and computational analysis, leveraging complex biological systems, multimodal sensors, and high-throughput methods to extract meaningful biological information. In this scenario, AI is transitioning from a specialised tool to a standard component of bioanalytical workflows. It supports the analysis of complex datasets, automates complex tasks, and augments human expertise by efficiently handling high-dimensional data and extracting information faster than traditional methods. Further, AI can uncover patterns in noisy, high-dimensional datasets, and identify non-obvious relationships, such as those between complex gene expression patterns and diseases, thereby bridging the gap between raw data and biological meaning.

This rapid integration of AI into bioanalytical workflows however represents more than a technological advance. From a higher education perspective, it fundamentally challenges how bioanalytics is taught and how students learn to interpret experimental data. AI methods, including machine learning and deep learning, are increasingly being integrated into routine analytical workflows, for example in spectroscopy, chromatography, imaging, omics, and sensor-based analyses. High-dimensional datasets from these approaches can now be translated into quantitative estimates of biological states with impressive accuracy, often with minimal user intervention. This situation can create a paradox for students: models may appear to work immediately and produce convincing numerical outputs, while the biological meaning of these results often remains unclear. A correct prediction does not necessarily imply understanding of the underlying biological system. Without explicit guidance, students may associate model performance with scientific insight, especially in analytical coursework, where correlation is easily mistaken for causality.

Despite the widespread use of AI in bioanalytical research, education has not yet caught up with this change, leading to a growing gap between research practice and teaching. The continued focus on small-scale, manual analysis, combined with the siloed structure of education in which biology and computer science are taught separately, further widens this gap. In addition, AI ethics currently receives limited attention in education, increasing the risk of flawed or unfair AI applications. As a result, graduates frequently lack the practical AI skills required in both industry and academia.

The use case described in this study illustrates that AI-supported environments place new demands on students: beyond purely procedural competence, they require the ability to critically evaluate results, recognise uncertainties, and relate computational results to biological and analytical plausibility.

In this context, the ‘grey-box’ concept presented in the “Educational implications and curriculum design” section offers a useful pedagogical perspective. Rather than treating subject knowledge as a layer of explanation added only after model predictions have been generated, grey-box approaches integrate biological, physical, and chemical assumptions directly into the modelling process itself. From a didactic perspective, this is important as it makes decisions in the modelling process more transparent and encourages students to investigate how predictions are arrived at. Students should therefore be repeatedly confronted with questions regarding which features of a signal actually influence a prediction, which patterns might represent artefacts, and how robust a conclusion remains under altered experimental conditions.

One possible curricular approach would be to structure learning in a step-by-step manner. Fundamental skills in analytical or bioanalytical chemistry would first need to be established independently of AI-based systems before more advanced computer-aided methods are introduced. These fundamentals should then reappear throughout the degree programme in increasingly complex contexts. Such a structure can help students to view AI outputs not as definitive results, but instead to situate them within broader analytical and scientific thought processes. Current discussions in higher education also argue that AI should complement, rather than replace, intellectual engagement and subject-specific understanding [1].

Within this framework, case-based and problem-oriented teaching formats take on particular relevance. Authentic datasets are rarely complete, clean, or unambiguous. Consequently, students in educational settings should also work with noisy or partially inconsistent data. Encountering imperfect data can stimulate more intensive reflection on model uncertainty, experimental limitations, and the interpretative limits of AI-supported analyses. In the life sciences in particular, research-based learning environments appear well-suited to fostering critical thinking, as they directly confront students with the gap between statistical prediction and biological interpretation. Under such conditions, the focus shifts away from merely arriving at correct answers towards constructing scientifically sound arguments.

The close link between laboratory practice and computer-assisted analysis should therefore become a central component of AI-oriented bioanalytical teaching. Teaching sequences could begin with manual data collection and conventional evaluation, before gradually introducing AI-assisted analyses and, finally, biological interpretation at a higher conceptual level. Going through these phases separately is pedagogically valuable, as it enables students to distinguish between observation, correlation, and mechanistic explanation, rather than combining these into a single automated workflow.

These changes also have implications for performance assessment. Traditional forms of assessment often emphasise efficiency and accuracy, whereas AI-supported scientific work increasingly depends on transparent reasoning, critical interpretation, and the ability to deal with uncertainty. Assessment strategies may therefore need to focus more on how conclusions are justified, how limitations are recognised and how confidently students can question AI-generated results. In particular, the identification of potential biases, methodological weaknesses, and context-dependent errors should be an explicit part of academic assessment, rather than remaining implicit. Current educational research also emphasises that scepticism, contextual judgement, and ethical awareness are essential competencies in AI-supported learning environments [1].

Issues of bias and data quality deserve explicit attention within the course itself. AI systems are shaped not only by training data, but also by decisions regarding pre-processing, model architecture, and underlying assumptions. Students must therefore understand how phenomena such as overfitting, data leaks, or algorithmic biases arise and why they are significant for scientific validity. Without such methodological awareness, there is a risk that computer-generated results may acquire unjustified authority despite potentially flawed empirical foundations.

Finally, AI in bioanalytical education should not be understood purely as a technical development. Issues of transparency, accountability, and social responsibility are directly linked to scientific practice and must not remain marginal topics. Universities therefore play a crucial role not only in imparting technical skills, but also in preparing students to act responsibly in increasingly complex and data-intensive research environments. Scientific integrity, critical judgement, and ethical reflection remain indispensable precisely because AI systems are gaining ever greater influence in analytical decision-making processes.

To address these challenges from both a scientific and educational perspective, this Perspective argues that AI-supported bioanalytics requires more than accurate prediction: model outputs must remain analytically valid, biologically interpretable, and educationally meaningful. We therefore discuss black-box, grey-box, and white-box modelling as complementary approaches to knowledge generation and examine their relevance for both research practice and higher education.

## From prediction to understanding: model transparency in bioanalytics

In traditional bioanalytical education, understanding is built on direct relationships between signal and substance. A spectral peak corresponds to a molecule, a calibration curve to a simple concentration-signal relationship. AI-based models can weaken this intuitive link. Predictions may succeed even when the biological interpretation of the signal remains hidden. This shift requires a deliberate pedagogical response. This is reinforced by the fact that, despite impressive technical progress, current AI-driven bioanalytics still faces challenges related to interpretability, robustness, and transferability [2, 3]. This situation calls for a stronger integration of model critique, uncertainty assessment and domain-specific interpretation into bioanalytical education. Students must learn not only how to build predictive models but how to question them. Which parts of the data drive the prediction? Which effects are biological, which physical and which artefacts of the measurement? Under which conditions might a model fail? In this sense, artificial intelligence does not reduce the need for analytical thinking, it amplifies it. The didactic challenge is therefore not to train better model users, but to develop reflective analysts capable of translating numerical predictions into meaningful biological insight [4, 5].

### Black-, grey-, and white-box models as knowledge-based paradigms

**Fig. 1:**
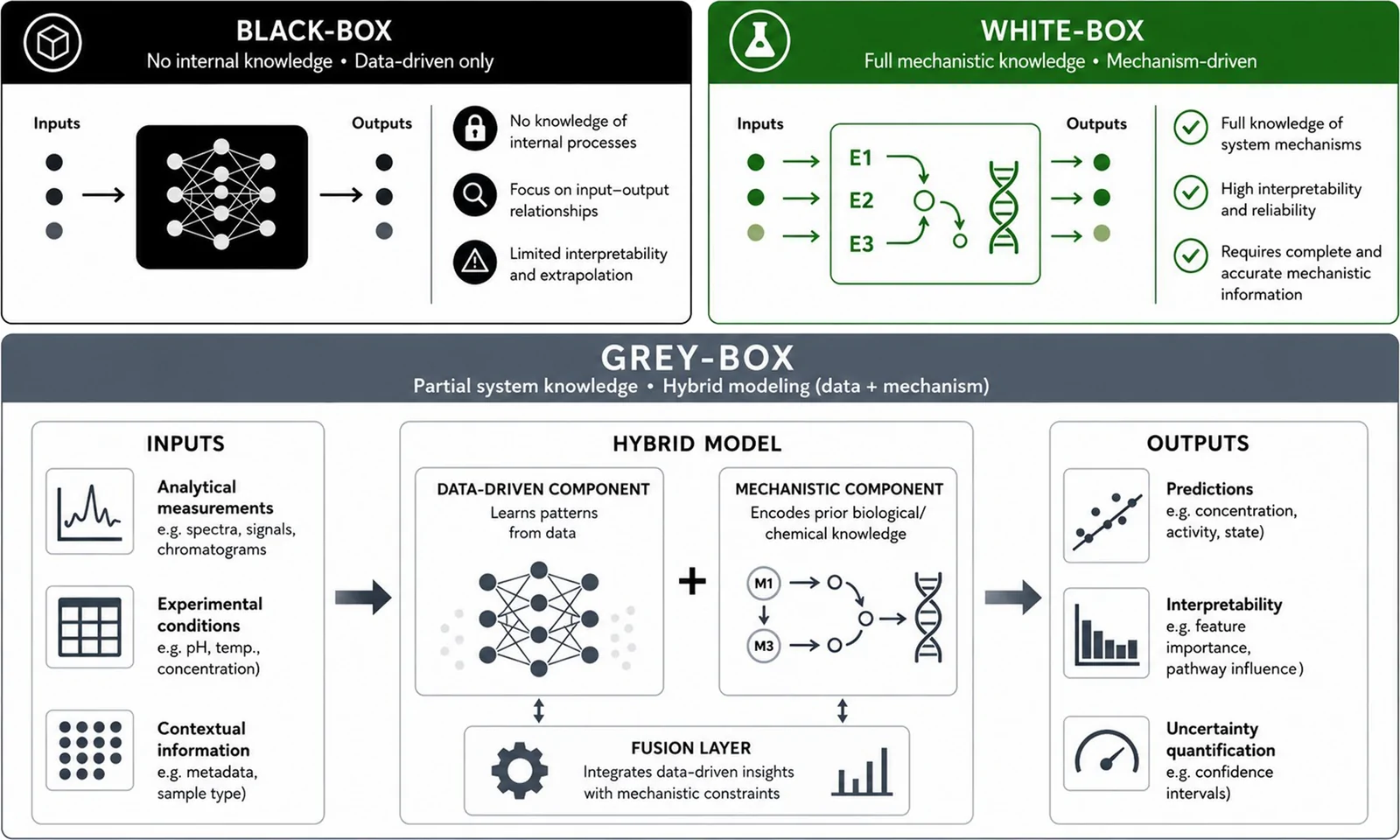
Conceptual framework of black-box, grey-box, and white-box modelling approaches in bioanalytical applications. The grey-box model is emphasised due to its role in integrating data-driven learning with mechanistic knowledge, thereby supporting both predictive performance and interpretability. Black-box models rely on empirical input-output relationships, while white-box models are fully mechanistic

Across systems engineering, control theory, and data-driven modelling, models are commonly classified according to their degree of transparency into black-box, grey-box, and white-box approaches (Fig. 1). While this distinction is often used in a purely technical sense, we argue that it is more productively understood as a knowledge-based framework reflecting fundamentally different modes of knowledge generation and interpretation. Models with similar predictive performance may therefore convey fundamentally different levels of understanding, depending on how assumptions, structure, and domain knowledge are embedded. In bioanalytics, this classification provides a useful framework for discussing how predictive performance, interpretability, and domain knowledge are balanced in analytical workflows.

Black-box models rely exclusively on input-output relationships learned from data, without explicitly encoding physical, chemical, or biological principles. In many scientific applications, this data-driven nature constitutes a pragmatic advantage, enabling high flexibility and strong predictive performance, particularly for complex, high-dimensional datasets where mechanistic descriptions are incomplete, unavailable, or impractical. Their primary limitation lies at the level of interpretability, as the internal decision logic is not directly accessible. Approaches from explainable artificial intelligence (XAI) [6] address this limitation by providing post-hoc or intrinsically interpretable representations of model behaviour, such as feature relevance, sensitivity patterns, or local decision structures. While XAI enhances transparency and supports model validation and hypothesis generation, it does not fundamentally change the data-driven nature of black-box models, as domain knowledge is used to interpret model outputs rather than to constrain model structure. White-box models, in contrast, are fully mechanistic and derive predictions from explicitly formulated equations representing known processes. While these models provide maximal transparency and causal interpretability, they often struggle to adequately represent complex biological systems in which relevant mechanisms are only partially understood or difficult to parameterise.

Grey-box models occupy the intermediate space between these paradigms. Rather than representing a single modelling technique, grey-box modelling constitutes a broader philosophy in which empirical data are interpreted through partial system knowledge. This integration may occur at different levels of abstraction, allowing known constraints, physical relationships, or biological plausibility to guide model development without enforcing a fully mechanistic formulation. Within this class, hybrid (semi-parametric) models form a more specific subset, characterised by an explicit architectural separation [7, 8]. Mechanistic equations define the model backbone, while data-driven components are confined to well-defined substructures such as rate expressions or closure relations. This distinction is not merely terminological, but directly affects how assumptions are encoded, how parameters can be interpreted, and how reliably models perform within their defined calibration space.

In bioanalytical applications, predictive performance is often treated as the primary metric of model quality. However, many analytical measurements rely on indirect observables where the measured signal is only loosely coupled to the biological or chemical quantity of interest. Under such conditions, accurate prediction does not necessarily imply scientific understanding. Purely data-driven models may capture stable correlations present in the training data while remaining insensitive to changes in experimental conditions, biological state, or measurement physics. This limitation becomes particularly critical when models are transferred across instruments, assays, or cultivation regimes, where previously learned correlations may no longer hold. As a result, prediction-centric workflows risk obscuring the distinction between correlation and causation [2, 8], especially when model outputs are interpreted without reference to underlying mechanisms. From an analytical standpoint, scientific validity therefore requires more than numerical agreement with reference data. It demands that predictions can be related to plausible physical, chemical, or biological processes and that deviations from expected behaviour can be meaningfully interpreted. Grey-box and hybrid modelling approaches address this requirement by embedding domain knowledge directly into the modelling framework, thereby constraining learning, reducing spurious correlations, and supporting extrapolation [7, 9]. In this sense, interpretability is not an auxiliary feature, but a prerequisite for robust analytical reasoning.

### Model transparency as a requirement for analytical validity and education

Beyond methodological considerations, model transparency also represents an educational challenge. When predictive models operate as opaque systems, responsibility for interpretation is implicitly shifted from the analyst to the algorithm, increasing the risk that numerical output is mistaken for genuine insight. Studies from educational research [4] highlight that black-box AI practices can foster a false sense of understanding, where learners focus on predictive success while the underlying reasoning remains elusive. Similar structural challenges have been identified in analytical chemistry education, where laboratory instruction often prioritises procedural correctness over interpretative understanding [5]. Students may execute complex analytical protocols successfully while remaining unaware of the assumptions, limitations, and decision points underlying their measurements. Approaches such as flipped laboratory models explicitly address this theory-practice gap by embedding justification, reflection, and conceptual activation into experimental workflows. Rather than emphasising execution alone, these frameworks encourage students to articulate why specific analytical choices are made and how results should be interpreted.

From this perspective, transparency in analytical modelling and transparency in laboratory education follow the same underlying principle. Scientific validity does not emerge from correct results alone, but from the ability to relate observations to underlying reasoning. AI can support this process when used as a scaffold for structured interpretation and reflection, rather than as a substitute for analytical judgment. By explicitly distinguishing between black-, grey-, and white-box paradigms, both research and teaching can move beyond prediction-centric practices toward models that genuinely support understanding [4, 7].

## Teaching case: physically informed data-driven monitoring of mammalian cell viability by UV/Vis spectroscopy

This case study illustrates how students can progress from an initially black-box use of a predictive chemometric model to a physically and biologically informed interpretation of UV/Vis spectra acquired during mammalian cell cultivation. The example is based on the experimental study reported by Drieschner et al. [10]. It is presented here as a structured teaching sequence rather than as a new experimental study. The underlying experimental data were obtained previously, and the educational workflow deliberately begins with an apparently successful prediction before asking what information the model uses, whether that information is specific to cell viability and under which conditions the prediction remains valid.

In this teaching case, AI is discussed in the broader context of data-driven methods that support the extraction, prediction and interpretation of information from complex bioanalytical data. The underlying experimental study applied partial least squares (PLS) regression and multivariate curve resolution-alternating least squares (MCR-ALS), two established chemometric approaches that share central principles with machine learning methods, including latent-variable modelling, pattern recognition, and data-driven analysis. PLS is initially used to obtain a supervised prediction of cell viability from the spectra. At this stage, the model is treated in a black-box manner because predictive performance is considered before the physical origin and specificity of the predictive information have been established. The subsequent controlled experiment and MCR-ALS analysis then introduce experimental and process knowledge and move the interpretation towards a grey-box perspective. After completing the case study, students should be able to differentiate between absorption-related and wavelength-dependent light scattering contributions in UV/Vis spectra of mammalian cell suspensions and explain why cell viability cannot be assigned to a single isolated spectral feature. They should be able to identify potential confounding between cell concentration, medium composition, cultivation time, and cell viability and to distinguish the predictive function of PLS regression from the component-resolving and interpretative function of MCR-ALS. Furthermore, students should learn to interpret MCR-derived profiles in relation to viable cells, non-viable cells, and medium-related contributions while recognising the model-based nature of these assignments. Finally, they should be able to evaluate predictive performance, model robustness, and analytical and biological plausibility as complementary aspects of model validity.

### Analytical challenge and teaching focus

Cell viability is a critical process parameter in mammalian cell cultivation, biopharmaceutical production, and cell-based assays. Reference methods such as dye-exclusion counting provide direct information but require sampling and therefore deliver only discrete off-line measurements. UV/Vis spectroscopy enables continuous and label-free spectral acquisition. However, the measured apparent absorbance is an indirect and composite signal rather than a direct measurement of cell viability. The spectra contain at least three overlapping sources of variation. First, the cultivation medium and dissolved or released cellular compounds contribute molecular absorption, particularly in the UV region. Signals around 230 nm are strong but chemically non-specific, while the region between approximately 260 and 280 nm may contain contributions from nucleic acids, proteins, and aromatic amino acids. In an intact cell suspension, these spectral features cannot be assigned to a single analyte without additional evidence. Second, cells and cell fragments scatter light. This contribution appears as a baseline offset and a wavelength-dependent spectral slope and depends on cell number, size, morphology, aggregation, and structural integrity. Third, these contributions change simultaneously during cultivation. Consequently, a correlation between a spectrum and viability does not demonstrate that the model has identified a viability-specific mechanism. This ambiguity constitutes the central analytical and didactic problem of the teaching case. Students are asked to distinguish three questions: Can cell viability be predicted? Which spectral information contributes to the prediction? Is this information sufficiently specific and robust to support an analytically meaningful interpretation?

#### Step 1: Inspecting the spectra and formulating initial hypotheses

**Fig. 2:**
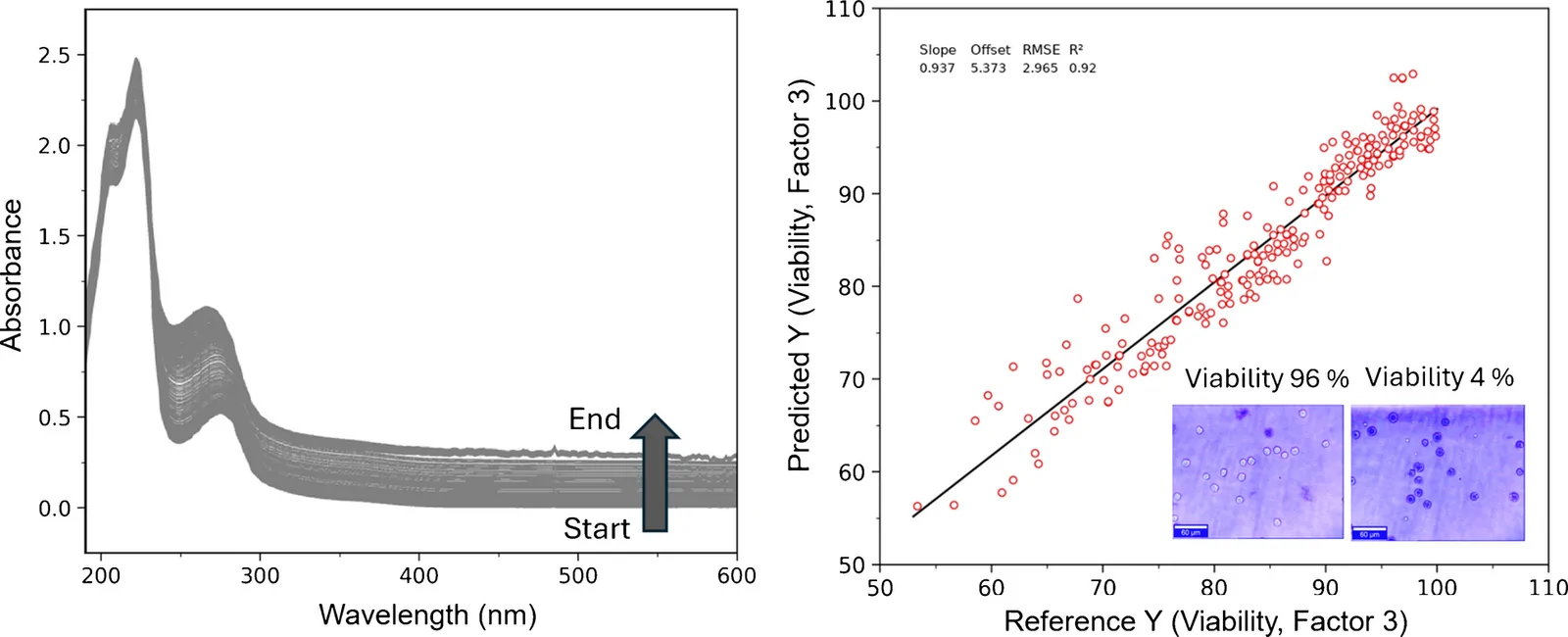
UV/Vis-based prediction of CHO K1 cell viability. Left: Apparent-absorbance spectra recorded from 200 to 600 nm using a TIDAS 1961 SSG spectrometer with a 1-mm immersion probe. No spectral pre-processing was applied. Right: Reference viability determined by trypan blue counting versus PLS-predicted viability using three latent variables. The inset shows representative cell suspensions at 96% and 4% viability

Before applying a multivariate model, students inspect the measured spectra and formulate initial, testable hypotheses about the origin of the observed spectral changes (Fig. 2, left). They identify pronounced UV features, baseline offsets, and changes in spectral slope and consider whether these observations may be related to molecular absorption, wavelength-dependent light scattering, or a combination of both. For example, an increase in apparent absorbance may result from changes in absorbing medium or cellular components, a higher total cell concentration, increased scattering by cells or cell fragments, or several of these effects occurring simultaneously. At this stage, students distinguish observation from interpretation and record which explanations require further evidence.

This first step anchors the analysis in the measurement and its underlying physical principles. UV/Vis spectroscopy alone cannot uniquely demonstrate specific biological processes such as apoptosis, membrane blebbing, or cellular fragmentation. Changes in spectral slope may be consistent with viability-dependent alterations in cell morphology and light scattering, but their biological interpretation requires support from reference measurements, controlled experiments, or microscopy.

#### Step 2: Building and critically examining an initial predictive model

A PLS regression model is used to predict reference cell viability from the full UV/Vis spectra (Fig. 2, right). PLS extracts latent variables that maximise the covariance between spectral variation and the reference viability values. Using three latent variables, the model provides good agreement between predicted and reference viability within the investigated application domain. Students evaluate slope, offset, RMSE, and *R*^2^ and, if the complete dataset is available, inspect the prediction residuals. A comprehensive model assessment should also include the PLS regression coefficients and, where appropriate, the weights and loadings of the latent variables, as these indicate which spectral regions contribute to the prediction. These parameters are not shown in the present teaching case. Moreover, their interpretation is often difficult for complex spectral data because neighbouring wavelengths are highly collinear and absorption and scattering contributions overlap. Figure 2 demonstrates that a convincing prediction can be obtained. Nevertheless, the analysis remains functionally black-box at this stage because neither the physical origin nor the specificity of the predictive information has yet been established. PLS is a powerful and comparatively transparent modelling approach rather than an inherently black-box method. However, its interpretative potential must be actively used instead of relying solely on predictive performance.

#### Step 3: Challenging the prediction through controlled perturbation

**Fig. 3:**
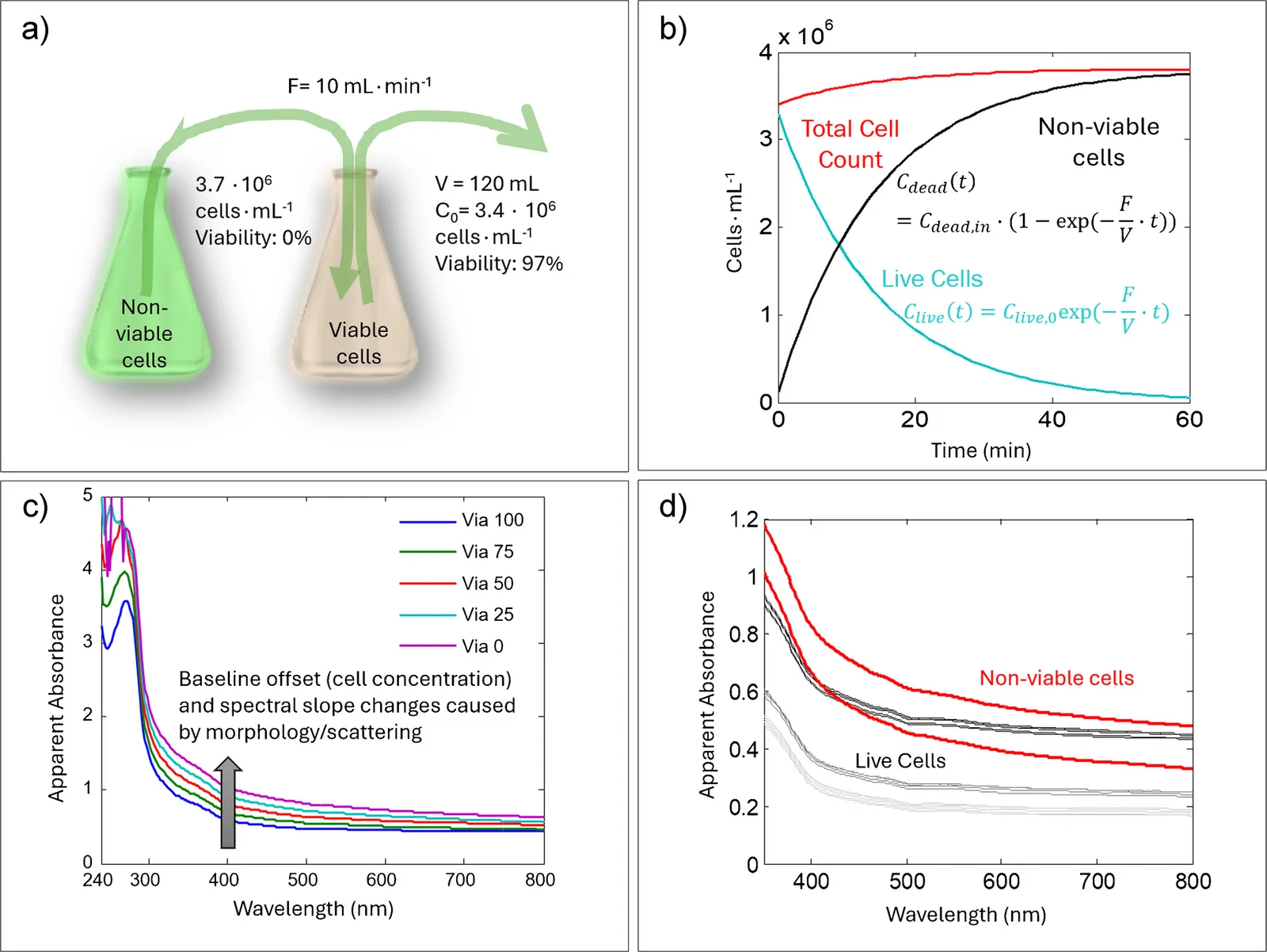
Inverse cultivation for separating viability- and cell-concentration-related spectral effects. **a** Non-viable cells were continuously added at 10 mL min⁻^1^ while an equal volume was removed to maintain 120 mL. **b** Cell concentrations calculated using the mixing model: viable cells (cyan), non-viable cells (black), and total cells (red). **c** UV/Vis spectra at viabilities of 100% (blue), 75% (green), 50% (red), 25% (cyan), and 0% (magenta), showing changes in baseline offset and spectral slope. **d** Comparison at similar apparent absorbance: red curves represent non-viable-cell suspensions, whereas black and grey curves represent viable-cell suspensions. The remaining spectral differences are consistent with viability-dependent changes in light scattering and cellular morphology

To move beyond the initial black-box result, students examine whether the PLS prediction is based on viability-related information or on correlated changes in total cell concentration, medium composition or cultivation time. The experimental study addressed this question using an inverse-cultivation approach [10]. During the simulated death phase, a suspension containing non-viable, starved cells was continuously introduced into a predominantly viable cell suspension, while an equivalent volume was removed to maintain a constant total volume (Fig. 3a). The resulting transition from predominantly viable to predominantly non-viable cells was described using a mixing model (Fig. 3b). The concentration of viable cells decreased continuously, whereas the concentration of non-viable cells increased. The total cell concentration remained within a comparatively narrow range, although it was not perfectly constant. This controlled perturbation reduced the confounding between cell viability and total cell concentration that occurs during conventional batch cultivation, in which viability, biomass, medium composition, and cultivation time typically change simultaneously. The spectra show changes in baseline offset, spectral slope, and band shape as viability decreases (Fig. 3c). The offset remains influenced by total cell concentration, whereas differences in spectral slope and shape provide additional information consistent with viability-dependent changes in cellular morphology and light scattering. Comparison of viable- and non-viable-cell suspensions at similar apparent-absorbance levels further supports systematic spectral differences between the two cellular states (Fig. 3d). However, these differences should not be interpreted as direct evidence of a specific cell-death mechanism.

Students use Fig. 3 to identify which variables were deliberately controlled and which remained potential confounders. They compare spectra of viable- and non-viable-cell suspensions at similar apparent-absorbance levels and reconsider the hypotheses formulated in Step 1. Differences that persist after approximate control of total cell concentration provide stronger evidence for viability-dependent scattering or morphological effects than correlations observed during an uncontrolled cultivation trajectory. The experiment thus turns a question raised by the predictive model into a testable analytical problem.

#### Step 4: Moving towards process-informed interpretation

**Fig. 4:**
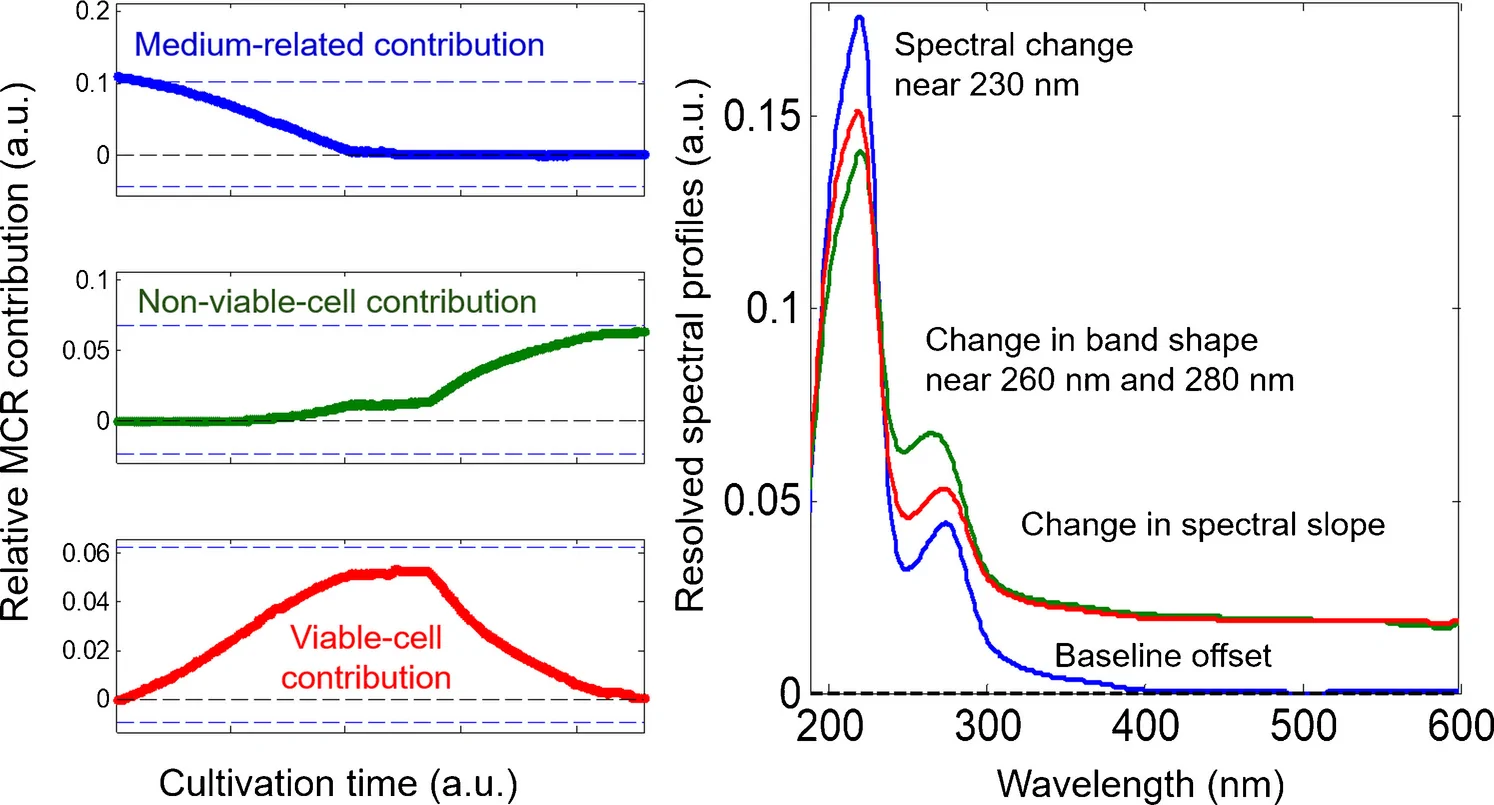
Three-component MCR-ALS decomposition of the UV/Vis spectra. The model was initialised using the purest-samples approach with non-negativity constraints on contribution and spectral profiles. Left: Relative normalised contributions assigned to medium-related changes, non-viable cells, and viable cells. Right: Corresponding resolved spectral profiles. Assignments were based on the cultivation trajectory, calculated cell profiles, and reference spectra and represent process-informed model contributions rather than absolute concentrations

The controlled experiment provides evidence that the spectral variation contains overlapping contributions, but it does not resolve them directly. MCR-ALS is therefore used as a complementary decomposition method. Conceptually, MCR-ALS decomposes the spectral data matrix into time-dependent contribution profiles and their corresponding resolved spectral profiles, together with a residual matrix. Unlike PLS regression, which relates the spectra directly to a reference variable, MCR-ALS aims to resolve coexisting spectral contributions that can subsequently be interpreted using experimental and process knowledge. In the present case, the resolved profiles are interpreted in relation to viable cells, non-viable cells, and medium-related changes (Fig. 4). This assignment is not generated automatically by the model. It is supported by the known progression of the cultivation, the calculated viable- and non-viable-cell profiles, the controlled experimental design and the characteristic spectral differences between viable- and non-viable-cell suspensions. The resulting MCR-ALS profiles represent relative, normalised model contributions in arbitrary units and not independently measured absolute concentrations. The medium-related profile represents spectral changes associated with alterations in medium composition during cultivation; it cannot be assigned to the concentration or consumption of a single identified medium component.

This step moves the analysis from an initially black-box use of prediction towards a grey-box perspective. MCR-ALS is not a mechanistic model, because it does not calculate light scattering from cell-size distributions or molecular absorption from known component concentrations. Nevertheless, its resolved profiles can be evaluated against the controlled experimental design, the cultivation trajectory, reference measurements, and measurement physics. The component assignments should therefore be understood as process-informed, model-based interpretations rather than as direct molecular or mechanistic identifications.

#### Step 5: Integrated validation and analytical judgement

The final evaluation deliberately asks whether the initially convincing result in Fig. 2 remains credible when predictive performance, experimental evidence, and physical interpretation are considered together. The allocation of experimental runs to calibration and independent external validation is defined before model fitting to prevent information leakage. Students assess the PLS model using slope, offset, RMSE, *R*^2^, and, if the complete dataset is available, prediction residuals. They distinguish internal model evaluation from performance on the independent external-validation runs and examine whether the magnitude or direction of the errors changes systematically across the investigated viability range. The MCR-ALS results are evaluated using complementary criteria. Students assess whether the resolved contribution profiles are consistent with the expected experimental trajectory and whether their spectral characteristics can plausibly be related to medium-related changes, viable cells, and non-viable cells. If spectral reconstruction and residual data are available, they also examine whether the model reproduces the measured spectra without systematic residual structure. Model validity is therefore assessed at three levels: numerical performance within the investigated application range, robustness under controlled changes in cell concentration and viability, and consistency with known physical and biological behaviour. Good prediction alone is treated as necessary but insufficient evidence. Disagreement between these criteria is used to identify limitations, potential confounding, and conditions under which an apparently accurate model may not be transferable.

### Discussion: What counts as AI in this case?

**Fig. 5:**
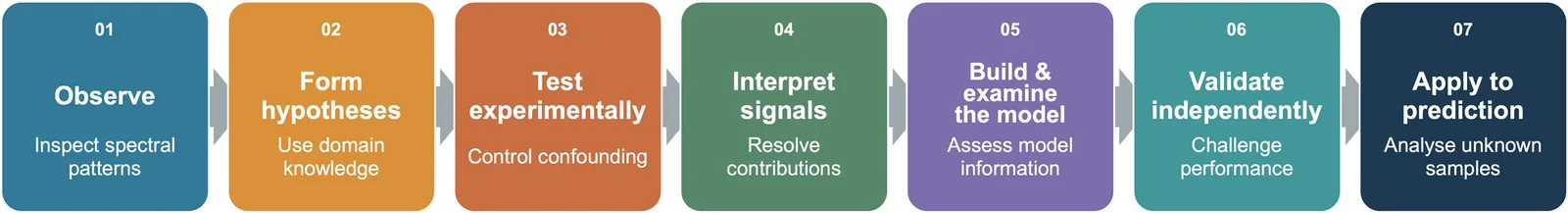
Recommended workflow for scientifically grounded prediction, derived from the teaching case. Spectral observation, hypothesis formation, controlled experimentation, and signal interpretation precede model development, independent validation, and application to unknown samples. Inconsistent evidence requires reconsideration of the hypotheses, experimental design, or model

The relevance of AI in this teaching case does not arise from the novelty of the algorithms employed. Rather, it lies in using multivariate models to learn relationships from complex spectral data and in critically examining how these relationships are translated into predictions and scientific interpretations. PLS and MCR-ALS are established chemometric methods that share important principles with machine learning, including latent-variable modelling, pattern recognition, and data-driven inference. Their comparative transparency and analytical accessibility make them particularly suitable for teaching both the potential and the limitations of AI-supported bioanalytical data analysis. The central lesson of the teaching case is the distinction between model performance and model understanding. An apparently successful prediction can provide a useful starting point, but it does not establish why the model works, whether the underlying information is specific to the target variable, or whether the observed relationship will remain valid under changed conditions. Model parameters such as regression coefficients, weights, and loadings can support this investigation, but they cannot by themselves provide an unequivocal physical, chemical, or biological explanation. This is particularly relevant for spectroscopic data, in which correlated wavelengths and overlapping absorption and scattering effects complicate interpretation. Figure 5 translates this lesson into a recommended workflow for future bioanalytical investigations. Students begin by inspecting the measured data and identifying reproducible spectral patterns without assigning them prematurely to a biological process.

They then formulate competing hypotheses based on measurement physics, chemical composition, and biological knowledge. Controlled experiments are used to vary relevant factors systematically, separate the target effect from potential confounders, and determine which hypotheses remain plausible. Exploratory or component-resolving methods can subsequently be applied to relate the observed signal variation to the experimental evidence. Only after this analytical understanding has been developed is a predictive model constructed and critically examined. Calibration and validation data must be separated before model optimisation and the final model must be challenged using independent samples and predefined performance criteria. Application to unknown samples represents the final step. If experimental evidence, model behaviour, and scientific interpretation are inconsistent, the workflow returns to hypothesis formation, experimental design, or model development. Within this workflow, AI-supported methods have a clearly defined role. They help identify multivariate relationships, resolve complex patterns, and generate predictive models, but they do not determine whether these relationships are causal, analytically specific, or biologically plausible. These questions require experimental design, reliable reference measurements, and domain knowledge. The combination of data-driven modelling with such external evidence moves the analysis from a purely prediction-oriented use towards a grey-box perspective. As an optional educational extension, the validated PLS model may be compared with a non-linear machine learning method. The comparison should address not only predictive accuracy but also robustness, interpretability, calibration-domain dependence, and consistency with measurement physics and biological knowledge. If no additional machine learning model was applied to the original dataset, this comparison should be explicitly identified as an educational extension. This case-specific discussion illustrates a broader educational principle: predictive accuracy alone does not constitute scientific understanding. The curricular implications of integrating AI-supported modelling with experimental design, measurement physics, and biological interpretation are discussed in the following section.

## Educational implications and curriculum design

The use case presented in this article illustrates a broader challenge for bioanalytical education. Students can achieve impressive predictive results without necessarily understanding the biological, chemical, or physical basis of these predictions. Consequently, the educational value of AI does not primarily lie in teaching students how to operate increasingly sophisticated modelling tools. Instead, it lies in helping them develop the ability to critically interpret results, evaluate their plausibility, and relate them to existing domain knowledge. From a curricular perspective, AI should therefore be embedded within existing bioanalytical learning environments rather than being taught as an isolated subject. Analytical chemistry, spectroscopy, experimental design, statistics, and biological interpretation remain the foundation upon which computational methods can be meaningfully applied. The aim is not to replace established scientific reasoning, but to extend it to increasingly data-rich analytical contexts.

The viability-monitoring example demonstrates that meaningful interpretation depends on more than model performance alone. Students benefit from learning environments in which experimental design, data acquisition, exploratory analysis, modelling, and validation are treated as interconnected elements of a single scientific process. In particular, the deliberate use of Design of Experiments helps learners understand how systematic variation can reveal causal relationships and expose confounding effects that may otherwise remain hidden in observational datasets. The distinction between black-box, grey-box, and white-box approaches provides a useful didactic framework for this purpose. Students should experience the strengths of purely data-driven models, but also their limitations. Equally important is the opportunity to connect model behaviour with established biological and physical knowledge. The educational value of grey-box approaches lies precisely in this integration. They encourage students to regard models not as sources of unquestionable answers but as scientific representations that require interpretation and validation. These learning objectives should also be reflected in assessment practices. Beyond technical correctness, students should be evaluated on their ability to justify methodological decisions, discuss uncertainties, identify limitations, and defend their interpretations. Reports, project work, oral presentations, and reflective assignments are therefore particularly well-suited to AI-oriented bioanalytical education because they make scientific reasoning visible.

Finally, the growing role of AI in analytical workflows reinforces the importance of scientific responsibility. Questions of data quality, transparency, bias, and appropriate interpretation should not be treated as peripheral topics but as integral components of professional bioanalytical practice. Graduates must be able not only to use computational tools effectively but also to judge when conclusions are scientifically justified and when additional evidence is required. In this sense, the educational challenge is not primarily technological but conceptual. The objective of bioanalytical education remains unchanged: to develop scientists who can transform observations into knowledge. The use case discussed in this article suggests that AI can support this goal most effectively when it is embedded within a teaching framework that consistently links prediction, interpretation, and scientific reasoning.

## Conclusions and outlook

Taken together, these considerations suggest that AI should not be treated as a separate addition to bioanalytical training, but rather as part of a broader scientific learning culture that prioritises reflection, interpretation, and critical questioning. The pedagogical relevance of AI lies not primarily in automation itself, but in how its use reshapes scientific thinking and the relationship between data, evidence, and explanation.

From this perspective, AI functions less as a substitute for scientific expertise and more as an extension of analytical capabilities. Machine learning systems are capable of identifying structures and correlations in highly complex datasets that would often remain inaccessible through conventional analysis alone. Yet predictive power is not automatically synonymous with understanding. In the life sciences in particular, there is a risk that statistically successful results will be mistakenly regarded as mechanistic explanations if the computational results are not interpreted within a suitable experimental and theoretical framework.

Educational research also shows that the integration of AI should be understood as a further development of existing teaching practices rather than a fundamental break with the past. Nevertheless, the increasing availability of generative and multimodal systems is changing the way students access information, formulate arguments, and construct knowledge. As AI-generated content becomes increasingly embedded in academic work, the ability to critically evaluate sources, assumptions, and interpretations is becoming correspondingly more important.

For this reason, the concept of AI literacy requires a broader interpretation.

Technical competence alone is not sufficient if students are unable to assess the reliability, limitations, and scientific implications of AI-generated results. A more reflective understanding of AI literacy would therefore include the ability to recognise uncertainties, identify potential biases, and evaluate how algorithmic systems shape scientific conclusions. Current debates in science education at university level are increasingly emphasising these competencies, particularly with regard to ethical responsibility and the contextual interpretation of data-driven results [11].

Under such conditions, the human scientist does not lose importance; rather, the focus shifts. Tasks such as interpretation, contextualisation, and ethical evaluation cannot simply be delegated to automated systems. On the contrary, they gain importance the more computationally complex the analytical workflows become. Scientific expertise increasingly depends not only on producing results, but also on assessing their validity, integrating them into broader knowledge structures, and recognising the limits of algorithmic thinking.

The interdisciplinary nature of AI-supported bioanalytics further reinforces this shift. Meaningful implementation requires collaboration between the natural sciences, computer-based disciplines, and ethical or societal perspectives. Study programmes should therefore promote interdisciplinary forms of collaboration, rather than treating technical and scientific competences as isolated fields. At the same time, the pedagogical integration of AI remains crucial. If AI is introduced primarily as a means of speeding up workflows or efficiently generating answers, students may perceive scientific research more as an optimisation process than as an investigative process. Used differently, however, AI can also deepen scientific understanding by encouraging students to question results, review assumptions and validate interpretations more carefully.

Ultimately, the future significance of AI in bioanalytical education will depend less on technological ingenuity than on the quality of the intellectual engagement with its use. The central challenge is therefore pedagogical rather than purely technical in nature. AI should promote the development of analytical judgement and scientific thinking, rather than limiting these to the passive acceptance of computational results.

## Data Availability

The datasets generated during and/or analysed during the current study are available from the corresponding author on reasonable request.
